# Bovine Colostrum Silage: Physicochemical and Microbiological Characteristics at Different Fermentation Times

**DOI:** 10.3389/fmicb.2021.708189

**Published:** 2021-09-13

**Authors:** Rosana Basso Kraus, Pedro Rassier dos Santos, Amanda Krummenauer, Kevin Eduardo Palhares, Helenice Gonzalez de Lima, Sílvia Regina Leal Ladeira, Giselda Maria Pereira, Giniani Carla Dors, Patrícia da Silva Nascente, Rafael Guerra Lund

**Affiliations:** ^1^Laboratory of Mycology and Bioprospecting, Post-Graduate Program in Biochemistry and Bioprospecting, Department of Microbiology and Parasitology, Biology Institute, Federal University of Pelotas, Pelotas, Brazil; ^2^Laboratory of Mycology and Bioprospecting, Post-Graduate Program in Microbiology and Parasitology, Department of Microbiology and Parasitology, Biology Institute, Federal University of Pelotas, Pelotas, Brazil; ^3^Residency Program in Veterinary Medicine, Faculty of Veterinary Medicine, Federal University of Pelotas, Pelotas, Brazil; ^4^Laboratory of Mycology and Bioprospecting, Department of Microbiology and Parasitology, Biology Institute, Federal University of Pelotas, Pelotas, Brazil; ^5^Department of Preventive Veterinary, Faculty of Veterinary Medicine, Federal University of Pelotas, Pelotas, Brazil; ^6^Regional Laboratory of Diagnostics, Faculty of Veterinary Medicine, Federal University of Pelotas, Pelotas, Brazil; ^7^Department of Mathematics and Statistics, Institute of Physics and Mathematics, Federal University of Pelotas, Pelotas, Brazil; ^8^Department of Science and Technology Agroindustrial, Faculty of Agronomy Eliseu Maciel, Federal University of Pelotas, Pelotas, Brazil

**Keywords:** bovine colostrum, silage colostrum, storage colostrum, bacteriological examination, quality

## Abstract

Bovine colostrum silage (BCS) is a technique used by milk producers for the conservation of bovine colostrum. However, it is necessary to ensure the safety and quality of BCS, as this food will be supplied to the animals. This study aimed to compare the physicochemical and microbiological compositions of colostrum silage at different fermentation times with milk and bovine colostrum (BC) quality parameters. BC samples were obtained from Jersey animals from one dairy farm. The BC samples (*n* = 21) were placed in 500-mL plastic bottles, stored vertically and anaerobically fermented for periods of 61–437 days. The following parameters of the physicochemical composition of the BCS were evaluated: acidity, protein, total solids and ash, using the methodologies of Adolfo Lutz Institute (2008). The microbiological analysis was developed according to the methodology proposed by Saalfeld et al. (2013), with adaptations. The acidity, total solids and protein over fermentation time (group 1: 61 to 154, group 2: 200 to 273, and group 3: 280 to 437 days) were not significantly different (*P* > 0.05). The ash content was significantly different (*P* < 0.05) in groups 1 and 3 and showed a decrease (moderate negative correlation of −0.63) with increasing fermentation time. Positive correlations were observed between total solids and the protein and ash contents. The genus of microorganisms with the highest occurrence was *Lactobacillus* spp. (95.2% of BCS) and those of lesser occurrence included *Escherichia* spp., *Actinomadura* spp., *Streptococcus* spp. and *Leuconostoc* spp. (4.8% of BCS). BCS has a physicochemical composition similar to BC and showed changes during the fermentation period; however, the presence of pathogenic microorganisms in BCSs reinforces the need to further explore the quality parameters for BCS to ensure the safety of animals who receive this food.

## HIGHLIGHTS

- *Lactobacillus* spp. were present in bovine colostrum silages for up to 437 days.

- This is the first study that compares the physicochemical parameters of milk and BC quality with colostrum silage.

- In this study, microbiological determinations were obtained from the fermentation of colostrum *in natura*.

- A non-significant correlation was found between the degree of acidity and the number of bacterial species.

## Introduction

Bovine colostrum (BC) is the first fluid produced postpartum (Solomons, 2002), and its main function is to provide immunity, increase physiological performance and provide better growth and development to newborn animals (Hurley and Theil, 2011). The composition of BC is superior when compared to the composition of milk in terms of the contents of lipids (BC: 4.6–6.7%; milk: 3.0%), protein (BC: 12.7–18.5%; milk: 3.1%), lactose (BC: 2.0–2.9%; milk: 4.5–5.2%), and total solids (BC: 14.1–27.2%; milk: 12.9%) (Morrill et al., 2012; Saalfeld et al., 2013; Mourad et al., 2014; Zarei et al., 2017). In addition, the presence of immunoglobulins, lactoferrins, lysozyme, leukocytes, cytokines and other immunomodulatory factors stands out. It is important to note that the physicochemical composition of BC differs according to the breed of animal and over the days in which it is excreted (Marnila and Korhonen, 2011; Morrill et al., 2012).

For many reasons, many newborn ruminants do not have access to their mothers’ colostrum immediately after birth: multiple births, acute mastitis, maladaptive maternal behavior mainly at first birth, etc., (Wereme et al., 2001). Therefore, it is necessary to create a colostrum bank for newborn calves that cannot be fed by their own mothers immediately after birth and for the preparation of immune colostrum products that can be used as food supplements or colostrum substitutes to provide effective protection against different enteric diseases in calves and humans (Alexieva et al., 2004).

In addition, dairy cows produce more colostrum than their own calves can consume (El-Fattah et al., 2014), and the concentrations of colostrum immunoglobulin (Ig), for example, are higher in the first postpartum milking and decrease rapidly over time (Alexieva et al., 2004). BC can be stored under refrigeration for up to 91 days (Ramya et al., 2016) to maintain chemical characteristics; however, this method of storage involves expenses with equipment and electricity consumption and affects the concentration of Ig.

Some methods for the preservation of BC have already been investigated: commercial sterilization, pasteurization, freezing, lyophilization (El-Fattah et al., 2014), vacuum evaporation, microwaving, spray drying (Chelack et al., 1993), refrigeration (Ramya et al., 2016), chemical preservatives (Muller and Smallcomb, 1977), drying (Borad and Singh, 2018), membrane filtration, ultrafiltration, microfiltration, high pressure (Gosch et al., 2014), pulsed electric field (Li et al., 2005), and irradiation (Pereira et al., 2014). These techniques have the advantage of preserving BC for a period of time ranging from 21 days to 3 months, but the main disadvantages are physicochemical changes, destruction of nutritional components (Borad and Singh, 2018), cost and the need for equipment.

The anaerobic fermentation of BC, called bovine colostrum silage (BCS), is a conservation method that has been studied and used (Ferreira et al., 2013; Saalfeld et al., 2013; Azevedo et al., 2014). This method allows BC storage for periods of up to 2 years; is a simple, low-cost process that does not require refrigeration, freezing or the use of additives; and can be used to feed calves (Saalfeld et al., 2013). In addition, BCS provides the transfer of immunoglobulin to calves in a similar manner to BC *in natura* (Saalfeld et al., 2014).

The objective of this study was to compare the physicochemical and microbiological compositions of colostrum silage at different fermentation times.

## Materials and Methods

### Bovine Colostrum

Bovine colostrum samples were obtained from Jersey animals (*n* = 21) from one dairy farm. The property is located in the city of Pelotas, state of Rio Grande do Sul, Brazil. The collections were carried out from March 2018 to April 2019. Table 1 shows the composition data and the somatic cell count (SCC) of milk obtained from the same animal after the end of colostrum production (International Dairy Federation, 2000, 2006).

**TABLE 1:** Physicochemical composition and somatic cell count of milk produced after BC by Jersey animals (*n* = 21) on a dairy farm located in southern Brazil in the city of Pelotas.

**Animals**	**Lipids**	**Protein**	**Lactose**	**Total solids**	**SCC**
A^1^					
B	4.06	3.05	4.45	12.48	25
C^2^					
D	3.61	3.05	4.99	12.61	67
E	3.16	3.00	4.70	11.79	271
F	3.73	4.04	4.12	12.88	6445
G	4.33	3.69	4.36	13.35	35
H^2^					
I	3.97	3.00	4.54	12.41	13
J	3.15	3.71	4.46	12.32	318
K	3.78	2.94	4.37	11.95	686
L	0.83	2.81	4.59	9.15	409
M	3.31	3.61	4.52	12.43	104
N^2^					
O	3.22	3.08	4.67	11.92	215
P^2^					
Q	4.25	3.46	4.27	12.91	12
R	3.88	3.26	4.68	12.76	35
S	3.64	3.10	4.40	12.03	15
T	3.71	3.53	4.62	12.84	175
U	3.14	3.55	4.42	12.05	21

*^1^Animal A died after giving birth.*

*^2^Data from animals C, H, N and P regarding composition and SCC could not be gathered from the producer.*

*BC, bovine colostrum; SCC, somatic cell count.*

### BC Silage

The BC samples (*n* = 21) were placed in 500-mL plastic bottles, stored vertically in a dry environment with natural light (light/dark), and anaerobically fermented for the period shown in Supplementary Table 1 (minimum of 61 days and a maximum of 437 days). Supplementary Table 1 also presents the animal information. Supplementary Table 2 shows the average temperature in each fermentation period.

### Sample Preparation for Physicochemical and Microbiological Analysis

Shortly after the fermentation period, the samples of BCS were removed from the containers, homogenized in a blender for 30 s, transferred to sterile 80-mL plastic containers and stored at −4°C until the time of analysis.

### Physicochemical and Microbiological Analyses

The following parameters of the physicochemical composition of the silage were evaluated: acidity (No. 427/IV), protein (No. 037/IV), total solids (No. 429/IV), and ash (No. 437/IV), using the methodologies of Adolfo Lutz Institute (2008). The physicochemical analyses were performed in duplicate.

The microbiological analysis was developed according to the methodology proposed by Saalfeld et al. (2013), with adaptations. BCS was sown in Chapman agar (Kasvi, Brazil), MacConkey agar (Himedia, France) and brain heart infusion agar (Kasvi, Brazil) as culture media. Thereafter, the plates were aerobically incubated at 37°C for 72 h. Then, the identification of isolated microorganisms was carried out using the Gram stain technique and biochemical tests (Barrow and Feltham, 1993). The presumptive identification of *Lactobacillus* spp. was performed on selective agar for *Lactobacillus* (Acumedia, United States), only observing the growth of these microorganisms.

### Statistical Analysis

The Kruskal–Wallis test, Dunn’s posttest and Spearman’s correlation analyses of variance were performed to determine the influence of the fermentation time (group 1: 61 to 154, group 2: 200 to 273, and group 3: 280 to 437 days) on the variables acidity, total solids, protein and ash. The Kruskal–Wallis test was used to assess the relationship between the acidity content (group 1: 7.5 to 19, group 2: 21 to 26.5, and group 3: 29 to 35°D) and the genera of the microorganisms identified. Statistical analyses were performed in BioEstat software and in the R environment, with a 95% significance level (*P* < 0.05).

## Results

### Physicochemical Analyses

Table 2 shows the physicochemical compositions of BCS at different fermentation times. The acidity contents ranged from 10.5 to 33.5°D, 11.5 to 35°D, and 8 to 30°D, with averages of 20.8, 22.7, and 20.8°D in groups 1, 2, and 3 (61 to 154, 200 to 273, and 280 to 437 days), respectively. The protein contents obtained ranged from 5.1 to 13.2%, 4.3 to 14%, and 3.5 to 13.7%, with averages of 10.9, 9.5, and 7.3% in groups 1, 2, and 3 (61 to 154, 200 to 273, and 280 to 437 days), respectively. The total solids reached percentages of 8.1 to 22.3%, 8 to 21%, and 6.8 to 19.5%, with averages of 16.7, 14.2, and 11.6% in groups 1, 2, and 3 (61 to 154, 200 to 273, and 280 to 437 days), respectively. The ash contents varied from 1.19 to 1.33%, 1.03 to 1.29%, and 0.45 to 1.27%, with averages of 1.29, 1.17, and 0.98% in groups 1, 2, and 3 (61 to 154, 200 to 273, and 280 to 437 days), respectively.

**TABLE 2:** Physicochemical composition of BCS resulting from the fermentation of BC of Jersey animals (*n* = 21) from one dairy farm located in southern Brazil in the city of Pelotas.

**Animals**	**BCS fermentation time (days)**	**Acidity (°D)^1^**	**Protein (%)^1^**	**Total solids (%)^1^**	**Ashes (%)^1^**
S	61	17.5 ± 2.1	13.1 ± 0.2	18.0 ± 0.2	1.32 ± 0.18
Q	78	10.5 ± 2.1	11.0 ± 0.1	22.3 ± 0.2	1.31 ± 0.27
R	104	19.0 ± 1.4	12.3 ± 0.2	16.3 ± 0.2	1.29 ± 0.13
U	148	23.5 ± 0.7	5.1 ± 0.1	8.1 ± 0.2	1.19 ± 0.17
T	154	33.5 ± 2.1	13.2 ± 0.4	19.0 ± 0.1	1.33 ± 0.23
C	200	11.5 ± 0.7	10.6 ± 0.5	14.1 ± 0.2	1.09 ± 0.13
N	220	15.5 ± 2.1	12.2 ± 0.8	17.1 ± 0.2	1.28 ± 0.06
P	220	24.5 ± 3.5	12.0 ± 0.2	16.1 ± 0.1	1.29 ± 0.14
K	233	26.5 ± 6.4	13.0 ± 1.1	19.8 ± 0.1	1.25 ± 0.06
L	245	21.0 ± 0.0	4.3 ± 0.1	9.1 ± 0.1	1.11 ± 0.16
O	247	18.5 ± 0.7	4.7 ± 0.3	8.0 ± 0.1	1.07 ± 0.08
G	262	35.0 ± 7.1	5.1 ± 0.8	8.6 ± 0.0	1.03 ± 0.16
H	273	29.0 ± 1.4	14.0 ± 0.7	20.9 ± 0.3	1.28 ± 0.25
E	280	21.0 ± 2.8	4.9 ± 0.2	8.7 ± 0.3	1.04 ± 0.06
I	293	26.5 ± 0.7	3.5 ± 0.7	6.7 ± 0.2	0.55 ± 0.07
F	303	8.0 ± 0.0	13.7 ± 0.6	19.5 ± 0.2	1.27 ± 0.17
J	306	21.0 ± 0.0	4.8 ± 0.1	7.9 ± 0.1	1.11 ± 0.16
D	311	26.5 ± 2.1	3.5 ± 0.8	7.0 ± 0.1	0.45 ± 0.07
M	314	25.5 ± 2.1	4.3 ± 0.3	8.95 ± 0.2	1.13 ± 0.16
A	380	7.5 ± 0.7	11.2 ± 1.1	14.7 ± 0.1	1.02 ± 0.04
B	437	30.0 ± 0.0	12.1 ± 0.2	19.1 ± 0.1	1.24 ± 0.13

*^1^Mean ± standard deviation.*

When analyzing the averages of the fermentation time groups, it was noted that the acidity increased until the fermentative time of group 2 (200–273 days), followed by a reduction in group 3 (280–437 days), and the percentages of protein, total solids and ash decreased over the fermentation time. The present work found variations in the acidity, protein, total solids and ash results, and this behavior may be related to the longer fermentation period.

When comparing the different fermentation time groups (group 1: 61 to 154, group 2: 200 to 273, and group 3: 280 to 437 days) for the variables acidity, total solids and protein, no significant differences were observed (*P* > 0.05) (Table 3), meaning that these components were not influenced by fermentation time. A significant difference was observed in ash content (*P* < 0.05) in groups 1 and 3 (61 to 154 days and 280 to 437 days, respectively), decreasing (moderate negative correlation of −0.63) with increasing fermentation time. When verifying the influence between the studied variables and the fermentation time (group 1: 61 to 154, group 2: 200 to 273, and group 3: 280 to 437 days), it was observed that the total solids (0.77) and protein percentage (0.76) were positively correlated with the ash content, indicating that as the ash concentration increased, so did the total solids and protein percentage. There was also a positive correlation (0.85) between protein concentration and the total solids: as the protein concentration increased, the total solids increased.

**TABLE 3:** Analysis of variance of protein, ash, total solids, and acidity concentrations at different BCS fermentation times.

**Parameters**		**DF**	**QS**	**MS**	***F***	***P***
Acidity	Treatment	2	18.23	3	0.14	0.87
	Residue	18	1207.77	2		
	Total	20	1226.00	1		
		DF	QS	MS	F	P
Protein	Treatment	2	45.21	3	1.40	0.27
	Residue	18	291.50	2		
	Total	20	336.71	1		
		DF	QS	MS	F	P
Total solids	Treatment	2	84.41	3	1.52	0.25
	Residue	18	499.10	2		
	Total	20	583.50	1		
		DF	QS	MS	F	P
Ashes	Treatment	2	0.33	3	3.91	0.04
	Residue	18	0.76	2		
	Total	20	1.09	1		

*BCS, bovine colostrum silage; DF, degrees of freedom; QS, quadratic sum; MS, medium squares; F, F value.*

*P < 0.05 is significant.*

When relating the physical–chemical composition of milk obtained after the production of BC with the parameters of milk quality through Normative Instruction numbers 76 and 77 of 2018 (BRAZIL, 2018a,b), it is possible to state that of the 21 milk samples, only one sample did not meet the requirements for the levels of lipids, protein and total solids; two samples did not present the minimum lactose concentration; and all samples were within the limits established for the SCC.

### Microbiological Analyses

Supplementary Table 3 identifies the microorganisms isolated in BCS at different fermentation times. *Lactobacillus* spp. Were isolated in 20 of the 21 silages; in addition, from 61 to 200 days of fermentation, the microorganisms identified were *Staphylococcus* spp., *Lactobacillus* spp., *Escherichia* spp., *Enterococcus* spp. And *Bacillus* spp. From 220 to 273 days, other genera of organisms appeared, such as *Corynebacterium* spp., and the genera *Staphylococcus* spp. and *Lactobacillus* spp. continued to grow. From 280 to 437 days, the organisms identified were *Actinomadura* spp., *Leuconostoc* spp., *Lactococcus* spp., and *Streptococcus* spp.; *Staphylococcus* spp., *Lactobacillus* spp. and *Corynebacterium* spp. still remained, and, again, the presence of *Bacillus* spp. and *Enterococcus* spp. was identified, as in the period from 61 to 200 days.

**FIGURE 1:**
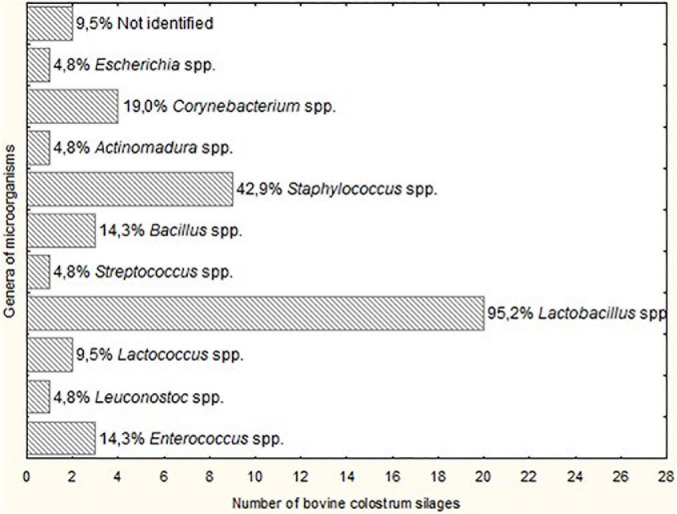
Genera of microorganisms isolated from Bovine colostrum silage (BCS) at different fermentation times.

The genus of microorganisms with the highest occurrence was *Lactobacillus* spp. (95.2% of BCS), and the genera of lesser occurrence were *Escherichia* spp., *Actinomadura* spp., *Streptococcus* spp. and *Leuconostoc* spp. (4.8% of BCS), as illustrated in Figure 1. There was a reduction in the number of genera of microorganisms isolated from BCS, but it was not possible to observe a significant difference (*P* > 0.05) between the acidity groups (group 1: 7.5 to 19, group 2: 21 to 26.5, and group 3: 29 to 35°D).

## Discussion

This study was the first to compare the physicochemical and microbiological parameters of milk quality with those obtained from BCS at different fermentation times. It is worth mentioning that the BC samples were acquired directly from milk producers and represent the handling performed in the daily lives of these producers. For this reason, it was decided not to carry out experimental planning with defined time and temperatures since the development of BCS in the properties occurs at room temperature and for as long as the producer needs. Aiming to reproduce the conditions carried out by the producers, another limitation was found in the present study in relation to the number of samples (*n* = 21).

### Physicochemical Analyses

Saalfeld et al. (2013) carried out BCS with 60 days of anaerobic fermentation, and the percentages of lipids, protein, dry matter and ash remained throughout the period that was evaluated. Azevedo et al. (2014) evaluated the influence of BC milking day on BCS fermentation, and the BC obtained from the second and third milkings showed a 90.5% BCS disposal rate due to the microorganisms from the BC that cause putrid appearance and odor, expansion or even rupture of the packaging due to the high production of gases. Therefore, improper fermentations favor the growth of pathogenic organisms, in addition to lactic acid bacteria, and the fermentation temperature has an effect on the degradation speed of some parameters, such as pH, lactic acid, acidity, and lactose. Higher temperatures (32.5°C) cause lower physical-chemical contents than room temperature (17.4 to 21.5 and 22.5°C) and benefit the growth of pathogenic microorganisms (Ferreira et al., 2013).

The behavior of wash concentration over the fermentation time was already expected and can be observed in fermentative processes that use milk, such as yogurt and fermented milk (Souza et al., 2013; Andrade et al., 2015). The studies in the literature on BCS used shorter fermentation times (Ferreira et al., 2013; Saalfeld et al., 2013); for this reason, the physicochemical composition did not behave similarly to the present study. In addition, fermentation temperature also influences physicochemical concentrations. A temperature of 32.5°C resulted in lower rates and greater reductions in pH, acidity and concentration of lactic acid; however, the storage of BC at 22°C or room temperature (17.4–21.5°C) resulted in higher concentrations of these components (Ferreira et al., 2013).

### Microbiological Analyses

Azevedo et al. (2014) reported the occurrence of *Enterobacteriaceae*, *Staphylococcus* spp. and fungi in some BCS fermented for 33 days at 25°C. Ferreira et al. (2013) observed lower counts of lactic acid bacteria and *Enterobacteriaceae* when fermentation was carried out at higher temperatures (32.5°C), and the ambient temperature (17.4–21.5°C) had a significant influence on the development of microorganisms in the BCS.

Our findings could not be compared with the results of Saalfeld et al. (2016) because according to the methodology used in that study, the colostrum was inoculated with the bacteria to check whether the fermentation of BC inhibited microbial growth, and in the present study, we isolated the bacteria from the newly fermented colostrum.

The presence of pathogenic organisms such as *Staphylococcus* spp., *Streptococcus* spp., and *Corynebacterium* spp. in the BCS must be limited; however, there is no legislation in force in Brazil for BC or BCS. Normative Instruction number 76 (BRAZIL, 2018a) limits bacterial and somatic cell counts for milk and can be used as a basis for the control of microorganisms in BC and BCS. Limitations were found regarding physicochemical and microbiological determinations due to the absence of standardized methodologies for BC and BCS, and for this reason, methods applied to milk were used.

There were some limitations in the present study, such as the sample size, which could impair the power of the statistical analysis; all samples of BCS were collected from one farm; and the absence of colostrum regulations, including specifications about the concentrations of microorganism genera that would be allowed in the silage as well as the variation of the fermentation time, had an influence on the microorganisms present.

## Conclusion

The physicochemical composition of BCS showed changes during the fermentation period. Nevertheless, pathogenic microorganisms were present at different times of fermentation. The presence of these organisms does not meet the milk quality parameters. Thus, it is recommended to use mild temperatures (17–22.5°C) for a minimum period of 35 days to carry out the silage. With regard to future perspectives, a course on good practices will be held, reinforcing cleaning and hygiene techniques before and after milking to reduce microbiological contamination of BCS.

## Data Availability Statement

The original contributions presented in the study are included in the article/Supplementary Material, further inquiries can be directed to the corresponding author/s.

## Author Contributions

RK contributed to antimicrobial evaluation and drafted the manuscript. PS and AK contributed to the physicochemical and microbiological analyses. KP contributed to the collection and identification of colostrum samples and microbiological analysis. HL and SL contributed to the conception of this study and supervised the laboratory work. GP contributed to the statistical analyses. GD and PN contributed to the design of this study and conception and the data interpretation. RL coordinated the study and contributed to critical reading of the manuscript. All the authors have read the final manuscript and approved the submission.

## Conflict of Interest

The authors declare that the research was conducted in the absence of any commercial or financial relationships that could be construed as a potential conflict of interest.

## Publisher’s Note

All claims expressed in this article are solely those of the authors and do not necessarily represent those of their affiliated organizations, or those of the publisher, the editors and the reviewers. Any product that may be evaluated in this article, or claim that may be made by its manufacturer, is not guaranteed or endorsed by the publisher.
