# Supplementary material for: Bovine Colostrum Silage: Physicochemical and Microbiological Characteristics at Different Fermentation Times
Source: Front Microbiol. 2021 Sep 13;12:708189. doi: 10.3389/fmicb.2021.708189 (PMC8473896; doi:10.3389/fmicb.2021.708189)
Supplement: Supplementary file 1 [file Data_Sheet_1.docx]

**Supplemental Table S1.** Description of animals according to the BCS fermentation time obtained from Jersey animals (n = 21) in dairy farm located in the South of Brazil, in the city of Pelotas

| Animals | Age of animals (years) | Date of delivery (day/month/year) | Number of deliveries | BCS fermentation time (days) | Annual fermentation period for BCS |
| --- | --- | --- | --- | --- | --- |
| S | 4 | 11/04/2019 | 2 | 61 | Autumn, winter |
| Q | 8 | 25/03/2019 | 6 | 78 | Autumn, winter |
| R | 4 | 27/02/2019 | 2 | 104 | Summer, autumn, winter |
| U | 6 | 14/01/2019 | 4 | 148 | Summer, autumn, winter |
| T | 8 | 08/01/2019 | 6 | 154 | Summer, autumn, winter |
| C | 4 | 16/11/2018 | 2 | 200 | Spring, summer, autumn, winter |
| N | 14 | 03/11/2018 | 12 | 220 | Spring, summer, autumn, winter |
| P | 8 | 03/11/2018 | 6 | 220 | Spring, summer, autumn, winter |
| K | 8 | 21/10/2018 | 6 | 233 | Spring, summer, autumn, winter |
| L | 7 | 09/10/2018 | 5 | 245 | Spring, summer, autumn, winter |
| O | 3 | 07/10/2018 | 1 | 247 | Spring, summer, autumn, winter |
| G | 3 | 15/09/2018 | 1 | 262 | Spring, summer, autumn, winter |
| H | 4 | 04/09/2018 | 2 | 273 | Spring, summer, autumn, winter |
| E | 5 | 28/08/2018 | 3 | 280 | Winter, spring, summer, autumn, winter |
| I | 4 | 15/08/2018 | 2 | 293 | Winter, spring, summer, autumn, winter |
| F | 9 | 05/08/2018 | 7 | 303 | Winter, spring, summer, autumn, winter |
| J | 10 | 02/08/2018 | 8 | 306 | Winter, spring, summer, autumn, winter |
| D | 5 | 28/07/2018 | 3 | 311 | Winter, spring, summer, autumn, winter |
| M | 5 | 01/08/2018 | 3 | 314 | Winter, spring, summer, autumn, winter |
| A | 6 | 20/05/2018 | 4 | 380 | Autumn, winter, spring, summer, autumn, winter |
| B | 5 | 24/03/2018 | 3 | 437 | Autumn, winter, spring, summer, autumn, winter |

^1^BCS – bovine colostrum silage.

**Supplemental Table S2.** Average fermentation temperature of BCS obtained from Jersey animals (n = 21) in dairy farm located in the South of Brazil, in the city Pelotas

| Seasons | Year / Month | Average monthly temperature (°C) | Average season temperature (°C) |
| --- | --- | --- | --- |
| Summer | 2017 / December  2018 / January  2018 / February | 22.5  23.7  22.4 | 22.9 |
| Autumn | 2018 / March  2018 / April  2018 / May | 20.7  21.7  16.4 | 19.6 |
| Winter | 2018 / June  2018 / July  2018 / August | 11.4  12.1  12.5 | 12.0 |
| Spring | 2018 / September  2018 / October  2018 / November | 17.3  17.5  21.2 | 18.7 |
| Summer | 2018 / December  2019 / January  2019 / February | 22.2  24.7  23.2 | 23.4 |
| Autumn | 2019 / March  2019 / April  2019 / May | 21.4  19.6  17.3 | 19.4 |

^1^Agroclimatology Station of Pelotas (EAPEL).

^2^BCS - bovine colostrum silage.

**Supplemental Table S3.** Identification of microorganisms isolated from BCS

| Animals | BCS fermentation time (days) | Microorganisms |
| --- | --- | --- |
| S | 61 | *Staphylococcus aureus*, *Lactobacillus* spp. |
| Q | 78 | *Escherichia* sp., *Staphylococcus* coagulase negative, *Enterococcus faecalis*, *Bacillus mycoides*, *Escherichia coli*, *Lactobacillus* spp. |
| R | 104 | *Lactococcus lactis*, *Lactobacillus* spp. |
| U | 148 | *Staphylococcus* coagulase negative, *Staphylococcus aureus*, *Lactobacillus* spp. |
| T | 154 | *Staphylococcus* coagulase negative, *Staphylococcus pseudointermedius*, *Lactobacillus* spp. |
| C | 200 | Not identified, *Bacillus* spp., *Staphycoccus* spp., *Lactobacillus* spp. |
| N | 220 | *Corynebacterium kutscheri*, *Lactobacillus* spp. |
| P | 220 | *Staphylococcus aureus* |
| K | 233 | *Lactobacillus* spp. |
| L | 245 | *Lactobacillus* spp. |
| O | 247 | *Staphylococcus aureus*, *Lactobacillus* spp. |
| G | 262 | *Staphylococcus* coagulase negative, *Lactobacillus* spp. |
| H | 273 | Not identified, *Lactobacillus* spp. |
| E | 280 | *Actinomadura madurae*, *Lactobacillus* spp. |
| I | 293 | *Corynebacterium* sp., *Corynebacterium amycolatum*, *Lactobacillus* spp. |
| F | 303 | *Corynebacterium*  sp., *Lactobacillus* spp. |
| J | 306 | *Corynebacterium* sp., *Staphylococcus pseudointermedius*, *Lactobacillus* spp. |
| D | 311 | *Bacillus pantothenticus*, *Bacillus* spp., *Lactobacillus* spp. |
| M | 314 | *Lactobacillus* spp. |
| A | 380 | *Enterococcus* sp., *Enterococcus faecalis*, *Leuconostoc* spp., *Lactococcus lactis*, *Lactobacillus* spp. |
| B | 437 | *Enterococcus durans*, *Streptococcus pyogenes*, *Lactobacillus* spp. |

^1^BCS - bovine colostrum silage.
